# Prognostic Factors in Necrotizing Fasciitis: Insights from a Two-Decade, Two-Center Study Involving 209 Cases

**DOI:** 10.3390/idr16030035

**Published:** 2024-05-16

**Authors:** Ioannis-Fivos Megas, Sarina Delavari, Alejandro Marti Edo, Götz Habild, Moritz Billner, Bert Reichert, David Breidung

**Affiliations:** 1 Department of Plastic, Reconstructive and Hand Surgery, Center for Severe Burn Injuries, Klinikum Nürnberg, Paracelsus Medical University, 90471 Nuremberg, Germany; ioannis.megas@jsd.de (I.-F.M.); sarina.delavari@martha-maria.de (S.D.); alejandro.martiedo@klinikum-nuernberg.de (A.M.E.); moritz.billner@klinikum-nuernberg.de (M.B.); bert.reichert@klinikum-nuernberg.de (B.R.); 2 Department of Orthopaedic and Trauma Surgery, Center of Plastic Surgery, Hand Surgery and Microsurgery, Evangelisches Waldkrankenhaus Spandau, 13589 Berlin, Germany; 3 Department of General and Visceral Surgery, Hospital Martha-Maria, 90491 Nuremberg, Germany

**Keywords:** necrotizing fasciitis, necrotizing soft tissue infection, mortality

## Abstract

Introduction: Necrotizing fasciitis (NF) is a critical disease with high morbidity and mortality rates that poses significant challenges in diagnosis and treatment. Prognostic factors for the clinical course of NF remain unclear and are currently under research. This study aims to identify such factors in a large cohort of patients which represents a major comprehensive investigation of prognostic factors for NF. Methods: Retrospective analysis was conducted on necrotizing fasciitis cases from 2003 to 2023 at two German hospitals. Data included demographics, comorbidities, laboratory findings, infection site, causative microorganisms and outcomes. Statistical analysis involved *t*-tests, chi-square tests, and ROC analysis. Results: A total of 209 patients were included, with a mortality rate of 18%. Patients were categorized into survivors (*n* = 171) and non-survivors (n = 38). Non-survivors were significantly older (68.9 ± 13.9 years vs. 55.9 ± 14.3 years; *p* < 0.01) and exhibited a higher prevalence of peripheral vascular diseases, cancer, and heart, liver, or renal insufficiency. Laboratory findings and scoring results also varied significantly between the two groups. The ROC curve analysis identified age as a predictor of mortality, with an optimal cut-off value of 68.5 years (sensitivity: 60.5%, specificity: 81.9%). Higher age was associated with increased mortality risk. Conclusions: The patient’s age stands out as the primary predictive element for mortality in necrotizing fasciitis. Additionally, we advocate for employing the Laboratory and Anamnestic Risk Indicator for Necrotizing Fasciitis (LARINF—score), which holds substantial prognostic significance and is straightforward to calculate. Considering our findings, crafting a clinical algorithm or scoring mechanism to forecast mortality in NF would be a promising target for future research.

## 1. Introduction

Necrotizing fasciitis (NF) is an emergency condition characterized by a high morbidity and mortality rate and poses a significant challenge in both diagnosis and treatment [1]. NF ranks among the most severe infections. The prevailing classification of NF delineates four primary types. Type 1 comprises mainly polymicrobial infections, which account for the majority of cases. Type 2 denotes monomicrobial infections caused by Gram-positive pathogens, whereas Type 3 involves Gram-negative pathogens, and Type 4 encompasses fungal infections [2,3]. Despite its rarity, necrotizing fasciitis should be considered as a possible diagnosis in various scenarios of soft tissue infections, as it progresses rapidly [4,5]. The importance of recognizing NF at an early stage is a well-known fact [6]. Several scoring systems have been developed over the years to help with initial decision making [4,7,8,9]. These have already been examined with regard to their reliability for diagnosis and also for prognostic accuracy [9,10,11,12,13,14]. Despite the importance of these decision-supporting tools in the acute situation, the prognostic factors for the further course of NF have not yet been conclusively clarified and are still the subject of current research [15,16,17]. The present study aims to determine prognostic factors in a large number of patients that are important for the clinical course of necrotizing fasciitis. For this purpose, two cohorts were examined and compared with each other: the survivors and the non-survivors. To our knowledge, our study is the largest cohort of patients ever investigated in Europe with regard to prognostic factors related to mortality in necrotizing fasciitis. By analyzing our extensive data set, we aim to gain crucial insights into the prognosis of necrotizing fasciitis and also make a significant contribution to the body of knowledge for clinical practice [15,16,18].

## 2. Materials and Methods

We conducted a retrospective analysis on patients diagnosed with necrotizing fasciitis at the Department of Plastic, Reconstructive, and Hand Surgery, Center for Severe Burn Injuries at Klinikum Nürnberg (Nuremberg, Germany) and the Department of Orthopedic and Trauma Surgery, Center of Plastic Surgery, Hand Surgery, and Microsurgery, Evangelisches Waldkrankenhaus Spandau (Berlin, Germany), including cases between 2003 and 2023. The patients included were identified on the basis of the International Classification of Diseases (ICD 10th revision) using the codes for necrotizing fasciitis (M72.6) and Fournier’s gangrene (N49.80 and N76.80). In addition, rare diseases such as Meleney’s gangrene were also included in our search; a combination of codes (L98.4/M72.6) was used. Our study included patients who presented with the full picture of acute necrotizing fasciitis and were treated specifically for this clinical condition. The data were collected from hospital records at the two centers, including demographics, comorbidities and risk factors, laboratory data, infection site, causative microorganisms and clinical outcomes such as hospital stay, intensive care unit (ICU) admission and mortality. The relevant laboratory data were generally the laboratory values taken upon admission to hospital; if necrotizing fasciitis developed during hospitalization, laboratory data were taken within 24 h prior to surgical intervention. Patients who were treated exclusively for defect reconstruction after necrotizing fasciitis and patients with solely necrotizing soft tissue infections (NSTI) were excluded.

Surgical debridement was prompted by a strong clinical suspicion of necrotizing fasciitis, supported by scoring systems such as the Laboratory Risk Indicator for Necrotizing Fasciitis (LRINEC) and the Laboratory and Anamnestic Risk Indicator for Necrotizing Fasciitis (LARINF) in recent years [4,7]. The LRINEC score, which was presented by Wong et al. in 2004, is an instrument for predicting the risk of necrotizing fasciitis. The score is based on six laboratory parameters: C-reactive protein, white blood cell count, hemoglobin, serum sodium, serum creatinine and glucose levels. While specific point allocations differ, each parameter contributes to the overall score, with C-reactive protein carrying the highest weight. Each parameter contributes points to the LRINEC score, ranging from zero to 13. Higher scores on the LRINEC indicate a greater risk of NF, with risk groups being assigned based on these scores. A score between zero and five indicates a low risk, a score of six or seven a moderate risk and a score of eight or more indicates a high risk of necrotizing fasciitis. The LARINF score presented by our research group in 2022 is also a diagnostic tool for evaluating patients for suspected necrotizing fasciitis and ultimately for the decision-making process for emergency surgical treatment. The score is based on the assessment of five parameters. These are hemoglobin, procalcitonin, C-reactive protein, heart, liver or renal insufficiency as a combined parameter, immunosuppression and obesity. Each parameter is also assigned a differently weighted point value. Scoring results between zero and 11 can be obtained, with a score of five or higher indicating an increased risk of necrotizing fasciitis. The diagnosis was confirmed histopathologically except in individual cases such as high suspicion and death before surgical intervention, or intraoperatively clinically conclusive findings of necrotizing fasciitis.

Data collection was performed using Microsoft^®^ Excel (Microsoft Corporation, Redmond, WA, USA) and analysis was performed with IBM^®^ SPSS^®^ Statistics (IBM Corp, Armonk, NY, USA). Data were checked for consistency and normal distribution. Statistical tests used were Student’s *t*-test and Pearson’s chi-square test. A two-sided *p*-value of <0.05 was defined as significant. The ROC curve was created with IBM^®^ SPSS^®^ Statistics (IBM Corp, Armonk, NY, USA).

## 3. Results

A total of 209 patients could be included in this study. One hundred seventy-six patients from Klinikum Nürnberg (Nuremberg, Germany) and thirty-three from Evangelisches Waldkrankenhaus Spandau (Berlin, Germany). The mortality rate in our study was 18%. An overview of the patients included in this study is given in Table 1.

To gain deeper insights into factors associated with survival in necrotizing fasciitis, we categorized patients into two groups: survivors (n = 171) and non-survivors (n = 38) (see Table 2). Significant differences emerged in several demographic parameters between survivors and non-survivors. Notably, non-survivors were significantly older than survivors (68.9 ± 13.9 years vs. 55.9 ± 14.3 years; *p* < 0.01). Gender distribution, however, did not differ significantly between the two groups (*p* = 0.30).

Comorbidities and risk factors played a crucial role in patient prognosis. Non-survivors exhibited a higher prevalence of peripheral vascular diseases (31.6% vs. 16.4%; *p* = 0.03), cancer (18.4% vs. 7.6%; *p* = 0.04) and heart, liver or renal insufficiency (81.6% vs. 41.5%; *p* < 0.01) compared to survivors. Additionally, hypertension (55.3% vs. 39.2%; *p* = 0.07), alcohol abuse (21.1% vs. 8.8%; *p* = 0.03) and drug abuse (2.6% vs. 9.9%; *p* = 0.21) were more prevalent among non-survivors. There were also notable differences between survivors and non-survivors with respect to the sites at which necrotizing fasciitis manifested. While infections of the lower extremities occurred more frequently in the non-survivors than in the survivors (60.5% vs. 39.2%; *p* = 0.03), no other statistically significant differences were found with regard to the localization of the infections (*p* > 0.05). Regarding the type of infections (polymicrobial infections vs. monomicrobial infections), there were no significant differences while regarding the Gram staining (Gram positive vs. Gram negative) of the monomicrobial infections was shown to be associated with the survival status of the patients (*p* = 0.04).

Laboratory findings provided further insights into prognostic factors. Non-survivors demonstrated significantly higher levels of creatinine (2.5 ± 1.9 mg/dL vs. 1.7 ± 1.6 mg/dL; *p* = 0.01) and glucose (137.7 ± 54.7 mg/dL vs. 188.7 ± 136.0 mg/dL; *p* < 0.01) compared to survivors. Scoring results also revealed differences between survivors and non-survivors. Non-survivors had higher LARINF scores compared to survivors (6.8 ± 1.6 vs. 5.9 ± 1.9; *p* = 0.02), indicating a greater severity of illness in this subgroup. Intensive care treatment was necessary in 152 patients, 38 of whom died which was shown to be statistically significance in terms of prognosis (*p* < 0.01).

**Figure 1:**
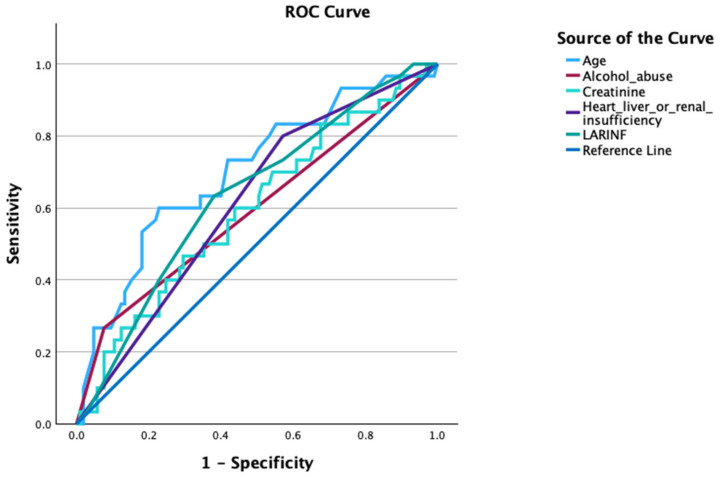
Receiver operating characteristic (ROC) curve for predicting mortality in patients with necrotizing fasciitis.

We performed a receiver operating characteristic (ROC) analysis with all demographic parameters, comorbidities and risk factors, laboratory data and scoring results. The five factors with the highest area under the curve are shown in Figure 1. The parameter of age revealed the highest area under the curve (0.72), followed by LARINF (0.63), heart, liver or renal insufficiency (0.61), creatinine (0.59) and alcohol (0.58). In the analysis of mortality prediction in patients with necrotizing fasciitis, age was evaluated as a potential predictor. The ROC curve analysis revealed that the optimal cut-off value for predicting mortality was determined to be 68.5 years, using Youden’s Index. It was observed that higher age was associated with an increased risk of mortality, with sensitivity and specificity at the optimal cut-off value estimated to be 60.5% and 81.9%, respectively.

## 4. Discussion

The words “life-threatening” and “mortality” are a recurring theme in the existing literature on necrotizing fasciitis [1,5,7]. Many researchers have been engaged in developing or evaluating existing scoring systems to aid in the diagnosis of NF [4,7,8,9,11,12,13,14,19]. However, the existing literature regarding the factors that are prognostic for the mortality of proven NF remains sparse. According to our literature search, there are only two other studies with a similar hypothesis with a cohort of more than 100 patients in addition to our study [15,18].

Our study showed an overall mortality rate of 18%, which is in line with the existing literature (12%; 22% and 32%) that also examined prognostic factors for NF and found an overall mortality rate [15,16,18]. However, the dispersion is striking and should be analyzed. The most notable observation is that the two largest studies (Kjaldgaard et al. and our study) have a lower average mortality. However, a mortality rate of 12% is surprising when one considers that the probable mortality rate for NF in the larger studies was estimated at 20–30% [13,14,15,20,21].

As our study shows, the age of the patients affected and the LARINF score have the highest prognostic value. The fact that the age of the patient plays a major role in the outcome of necrotizing fasciitis is hardly surprising. A large number of studies show this using the Physiological and Operative Severity Score for the enUmeration of Mortality and morbidity (POSSUM) scores for various clinical conditions, so it is not surprising that we obtained similar results when examining our cohorts (*p* < 0.01) [22,23]. This finding partly explains the differences in mortality between the two largest studies. Thus, when comparing the 12% mortality of Kjaldgaard et al. with the 18% mortality in our study, the mortality values are consecutively related to the average age of 50 versus 58 years [18].

The statistically significant prognostic value of the LARINF score (*p* = 0.02) also appears plausible, as it adds a number of clinical indicators (such as heart/liver/renal insufficiency/obesity and immunosuppression) to the laboratory indicators, which, when evaluated individually in other studies (>2 comorbidities), also yielded significant prognostic values [4,15]. This result is partially consistent with the results of van Stigt et al. In their study, which involved 123 patients, they concluded that the LRINEC score should be used for all patients with suspected necrotizing fasciitis and that older patients with two or more comorbidities have a higher mortality rate [15]. In addition, it must be mentioned that the LRINEC score is critically discussed and, among other research, no prognostic value could be determined in the context of NF in a recent study by our research group [11,19].

A closer look at the section on comorbidities reveals significant prognostic values for vascular peripheral diseases, such as peripheral artery disease (PAD) and chronic venous insufficiency (CVI) (31.6% vs. 16.4%; *p* = 0.03) and in the previously mentioned category of major organ insufficiency (81.6% vs. 41.5%; *p* < 0.01). This is also consistent with the existing literature, where significant values were also found for PAD, CVI and heart/liver/renal insufficiency [15,16,24].

Another parameter that proved to be prognostically significant for mortality was the need for perioperative intensive care treatment during the primary operation. As logical as this seems, it is also accurate for predicting mortality. For instance, the study by Abdalla et al. found a statistically significant correlation between perioperative intensive care treatment and mortality (51%; *p* < 0.001) [16]. Our study confirmed a significant association between the admission to an ICU and mortality. Based on our findings, it appears that approximately one-quarter of patients admitted to an intensive care unit with a diagnosis of necrotizing fasciitis do not survive.

The importance of identifying and accurately treating NF with antibiotics is unquestioned. As expected, monomicrobial infections were more frequent in both groups, but none of our analyses led to statistically significant values (*p* = 0.15). Only the further subdivision into Gram-positive and Gram-negative bacteria revealed statistically significant values among survivors within the Gram-positive group (*p* = 0.04). This is in line with the previous results of our research group, which identified Gram-negative infections as more dangerous, Gram-positive infections as more frequent, but also not statistically significant [3].

### 4.1. Strengths of the Study 

A strength of the present study lies in the use of a large patient cohort, making it the largest European study to date in the field of NF research. This extensive data set allows for a comprehensive analysis with the aim of identifying the perioperative clinical and laboratory parameters that have prognostic significance for mortality in NF cases. By utilizing this extensive pool of patient data, our study seeks to provide new insights into the prognostic landscape of NF and thus improve the understanding of this complex and potentially life-threatening disease. In this context, for example, we employed the LARINF score, which is an easy-to-use tool and in addition to its value in the diagnosis of NF, could now also have a prognostic value (*p* = 0.02). For everyday clinical practice, the findings of the present study could help to identify at-risk patients earlier and adapt treatment strategies accordingly, which could ultimately improve clinical care and patient survival.

### 4.2. Limitations of the Study

It is necessary to note the non-randomized and retrospective nature of the protocol used in this study, as this is a limitation that should be discussed. By using a non-randomized approach, biases may have influenced the selection and inclusion of patients in the cohorts studied. In addition, the retrospective design relies on data from the past, which may have certain limitations in terms of the accuracy and completeness of the data as well as potential confounding variables. The comparison of individual variables would also gain greater reliability within a prospective protocol, given that cohorts could be matched based on baseline characteristics. Another crucial aspect in the management of necrotizing fasciitis, the period of time between diagnosis and surgery, can only be accurately elucidated through a prospective study design. Furthermore, with pooled data from two centers, a certain bias from clinical handling must be assumed. However, this also underscores a strength of this study, as it contributed to the formation of a substantial patient cohort, which, according to our literature review, stands as one of the largest to date. Finally, a bias in overlapping prognostic factors must also be assumed, e.g., it is obvious that patients with necrotizing fasciitis of the lower extremity were also patients with PAD at the same time, both of which turned out to be statistically significant prognostic factors for mortality (both with a respective *p*-value of 0.03). 

## 5. Conclusions

The age of the patient is the most important prognostic factor for mortality in necrotizing fasciitis. We also recommend the use of the LARINF score, as this also has a significant prognostic value and is easy to determine. Based on our results, we believe that a prospective approach with the aim of developing a clinical algorithm or scoring system for the prognosis of mortality in NF is of interest for future research.

## Figures and Tables

**Table 1:** Baseline characteristics of the study population.

Parameters	Study Group (n = 209)
Demographics	
Age	58.3 ± 15.1
Male	139 (66.5%)
Comorbidities and risk factors	
Diabetes	85 (40.7%)
Peripheral vascular diseases	40 (19.1%)
Obesity	46 (22.0%)
Immunosuppression ^a^	13 (6.2%)
Cancer	20 (9.6%)
Heart, liver or renal insufficiency	102 (48.8%)
Hypertension	88 (42.1%)
Alcohol abuse	23 (11.0%)
Drug abuse	18 (8.6%)
Prior surgery	40 (19.1%)
Site of infection	
Upper Extremity	38 (18.2%)
Lower Extremity	90 (43.1%)
Cervical	3 (1.4%)
Trunk	17 (8.1%)
Genital	45 (21.5%)
Multiple Sites	16 (7.7%)
Causative Microorganisms	
Polymicrobial	73 (34.9%)
Monomicrobial	101 (48.3%)
Gram positive	67 (32.1%)
Gram negative	34 (16.3%)
No causative microorganism	35 (16.7%)
Laboratory findings	
C-reactive Protein, mg/dL	24.2 ± 23.6
Leukocyte count, per mm^3^	17.3 ± 13.1
Hemoglobin, g/dL	11.3 ± 2.3
Sodium, mmol/L	135.6 ± 5.9
Creatinine, mg/dL	1.9 ± 1.7
Glucose, mg/dL	179.4 ± 126.6
Procalcitonin ng/dL	14.4 ± 24.6
Scoring results	
Laboratory Risk Indicator for Necrotizing Fasciitis (LRINEC score)	6.1 ± 3.1
Laboratory and Anamnestic Risk Indicator for Necrotizing Fasciitis (LARINF score)	6.1 ± 1.9
Clinical outcome	
Length of hospital stay of survivors, days	35.6 ± 29.1
Admission to intensive care unit	152 (72.7%)
Number of deceased patients	38 (18.2%)

^a^: Diseases with a strong immunosuppressive impact, such as acquired immunodeficiency syndrome, and patients who are taking immunosuppressive drugs such as cortisone were counted as immunosuppression. Diabetes was not counted here as immunosuppression.

**Table 2:** Overview of patient characteristics, comorbidities, laboratory findings and scoring results.

Parameters	Survivors (n = 171)	Non-Survivors (n = 38)	*p*-Value
Demographics			
Age, years	55.9 ± 14.3	68.9 ± 13.9	<0.01
Male	111 (64.9%)	28 (73.7%)	0.30
Comorbidities and risk factors			
Diabetes	70 (40.9%)	15 (39.5%)	0.87
Peripheral vascular diseases	28 (16.4%)	12 (31.6%)	0.03
Obesity	36 (21.1%)	10 (26.3%)	0.48
Immunosuppression	11 (6.4%)	2 (5.3%)	0.79
Cancer	13 (7.6%	7 (18.4%)	0.04
Heart, liver or renal insufficiency	71 (41.5%)	31 (81.6%)	<0.01
Hypertension	67 (39.2%)	21 (55.3%)	0.07
Alcohol abuse	15 (8.8%)	8 (21.1%)	0.03
Drug abuse	17 (9.9%)	1 (2.6%)	0.21
Prior surgery	36 (21.1%)	4 (10.5%)	0.14
Site of infection			
Upper Extremity	35 (20.5%)	3 (7.9%)	0.06
Lower Extremity	67 (39.2%)	23 (60.5%)	0.03
Cervical	3 (1.8%)	0 (0.0%)	0.40
Trunk	15 (8.8%)	2 (5.3%)	0.72
Genital	36 (21.1%)	9 (23.7%)	0.50
Multiple Sites	15 (8.8%)	1 (2.6%)	0.34
Causative Microorganisms ^a^			
Polymicrobial	64 (37.4%)	9 (23.7%)	0.15
Monomicrobial	80 (46.8%)	21 (55.3%)	
Gram positive	57 (33.3%)	10 (26.3%)	0.04
Gram negative	23 (13.5%)	11 (28.9%)	
Laboratory findings			
C-reactive Protein, mg/dL	23.5 ± 22.9	27.5 ± 26.3	0.35
Leukocyte count, per mm^3^	16.7 ± 8.4	19.5 ± 25.3	0.52
Hemoglobin, g/dL	11.4 ± 2.4	11.1 ± 2.0	0.49
Sodium, mmol/L	135.5 ± 5.8	135.9 ± 6.1	0.76
Creatinine, mg/dL	1.7 ± 1.6	2.5 ± 1.9	0.01
Glucose, mg/dL	188.7 ± 136.0	137.7 ± 54.7	<0.01
Procalcitonin ng/dL	14.9 ± 26.3	12.6 ± 17.3	0.66
Scoring results and clinical outcome			
Laboratory Risk Indicator for Necrotizing Fasciitis (LRINEC score)	6.0 ± 3.2	6.5 ± 2.9	0.39
Laboratory and Anamnestic Risk Indicator for Necrotizing Fasciitis (LARINF score)	5.9 ± 1.9	6.8 ± 1.6	0.02
Admission to intensive care unit	114 (66.7%)	38 (100%)	<0.01

^a^: in a total of 35 patients, no causative microorganism could be identified.

## Data Availability

The data that support the findings of this study are available upon reasonable request from the corresponding author, [D.B.].
